# Comparison between single and three portal laparoscopic splenectomy in dogs

**DOI:** 10.1186/1746-6148-8-161

**Published:** 2012-09-10

**Authors:** Alireza Khalaj, Jalal Bakhtiari, Amir Niasari-Naslaji

**Affiliations:** 1 Minimally Invasive Surgery Research Centre, Rasool Akram Hospital, Tehran University of Medical Sciences, Tehran, Iran; 2 Department of Surgery, Faculty of Medicine, Shahed University, Tehran, Iran; 3 Faculty of Veterinary Medicine, University of Tehran, Tehran, Iran; 4 Department of Surgery and Radiology Faculty of Veterinary Medicine, University of Tehran, P. O. Box: 14155–6453, Tehran, Iran

## Abstract

**Background:**

Single incision laparoscopic surgery (SILS) is a newly growing technique to replace a more invasive conventional multiple portal laparoscopic surgery. The objective of this study was to compare single (SILS) with three portal (Conventional) laparoscopic splenectomy in dogs. Mongrel dogs (n = 18), weighting 15 ± 3 kg, were selected for this study (n = 12 SILS; n = 6 conventional). The area from xiphoid to pubis was prepared under aseptic conditions in dorsal recumbency with the head down and tilted 30 degree in the right lateral position. Pneumoperitoneum was established by CO_2_ using an automatic high flow pressure until achieving 12 mm Hg. Instrumentation used consisted of curved flexible-tip 5 mm Maryland forceps and ultracision harmonic scalpel for sealing and cutting of the vessels and splenic attachments.

**Results:**

All dogs recovered uneventfully. The splenectomy procedure using SILS and conventional methods were significantly different in the respective operative time (29.1 ± 1.65 *vs.* 42.0 + 2.69 min) and the length of the surgical scar (51.6 ± 1.34 mm *vs.* 72.0 ± 1.63 mm; P < 0.001). There were no post-operative wound complication including inflammation, infection, hernia formation and dehiscence up to one month after surgery. Meanwhile, the conversion to open surgery or application of additional portals was not required in both approaches.

**Conclusion:**

This study demonstrated that SILS is a safe and feasible operation and could be used as an alternative approach to three portal (Conventional) for splenectomy in dog.

## Background

The application of laparoscopy, minimally invasive technique, along with its advantages and superiorities, has become an alternative approach to conventional surgery in small animal veterinary medicine [1-3]. Laparoscopy created huge changes in the field of surgery from large incisions in open surgeries to very small incisions. Within the context of laparoscopy, single incision laparoscopy is a newly growing technique to reduce the invasiveness of conventional multiple portal laparoscopic surgery. Single incision laparoscopic surgery (SILS) reduces surgical trauma and adhesion through implementing a small number and/or size of portals [4-6]. SILS is a feasible technique for different ablative and reconstructive procedures such as cholecystectomy, appendectomy and splenectomy in human [7-10].

Splenectomy in dog is a common operation for tumors [11]. In human, hematological immune mediated diseases, non responsive to medical treatment, ITP and hemolytic anemia are the most common indications of splenectomy [12,13]. The ideal indication for the laparoscopic splenectomy is an elective splenectomy in blood donor dogs to prevent transmission of hemobartonella infection [13]. Clinical and experimental researches were conducted to elaborate laparoscopic and open splenectomy [14,15] resulting in the recognition of laparoscopy as a gold standard procedure for splenectomy [16,17]. In veterinary medicine, the feasibility of multiple portal laparoscopic splenectomy and its superiority over the conventional open technique was documented [11,18]. Conventional laparoscopy performed safely in laboratory animals, porcine, caprine, canine and human [18-21]. The objective of this study was to compare single (SILS) with three portal (Conventional) laparoscopic splenectomy in dogs.

## Results

Both SILS and conventional surgery were performed successfully and all dogs were recovered uneventfully. Splenic mobilization was successfully performed via a single umbilical incision. The final incision was extended for organ removal. There were significant difference (P < 0.001) between SILS and conventional surgery in the operative time (SLIS: 29.1 ± 1.65 min vs Conventional 42.0 ± 2.69 min) and the length of the surgical scar (SILS: 51.6 ± 1.34 mm vs Conventional: 72.0 ± 1.63 mm; Table 1). There was no significant difference in the length, diameter and weight of the spleen between two groups (Table 1). There were no post-operative complications including inflammation, infection, hernia formation and dehiscence up to one month after surgery. Slight and superficial rupture of spleen with very negligible and minor bleeding occurred in 4 cases (SILS: 3 dogs; Conventional: 1 dog) that was managed immediately without any particular requirement to perform open surgery or using additional portals to accomplish operation.

**Table 1:** Clinical and operative findings following splenectomy by single incision laparoscopic surgery (SILS; n = 10) and conventional (3 portals) laparoscopy (n = 6) in dogs

**Experimental groups**	**Operative time (min)**	**Scar length (mm)**	**Spleen**		
			**Length (mm)**	**Diameter (mm)**	**Weight (gr)**
SILS	29.1 ± 1.65^a^	51.6 ± 1.34^a^	28.9 ± 0.80^a^	10.1 ± 0.82^a^	275.4 ± 9.09^a^
Conventional	42.0 ± 2.69^b^	72.0 ± 1.63^b^	31.2 ± 0.87^a^	9.3 ± 1.56^a^	288.3 ± 6.14^a^

## Discussion

The purpose of this study was to investigate the possibility of replacing SILS with conventional standard 3 portals laparoscopy for splenectomy in dog. Accordingly, the operative time and scar length were found to be significantly less in SILS compared to conventional method. Single portal position in SILS provided similar visualization, manipulation and exposure of splenic hilum as in conventional laparoscopy. Insertion of single umbilical portal reduced the chance of accidental injury to splenic parenchyma compared to inserting three separate portals in conventional method.

Recently, the application of SILS techniques has been described in many intra-abdominal procedures in human [22]. The goal of single port access (SPA) surgery is to minimize the incision required to perform the procedure while maintaining the surgeon’s comfort [16]. To the authors’ knowledge, this is the first report in using SILS for splenectomy in dog. In general, less morbidity, short length of hospital stay, less post operative pain and excellent cosmetic results were considered as advantages of SILS to the conventional multiple portals laparoscopic surgeries [23]. Moreover, SILS splenectomy seems to be safe for intra operative visualization of the splenic hilum during transection of vessels and removal of spleen [24].

The operative time is an important parameter for surgical assessment. In the present study, the operative time was shorter in SILS (29.1 ± 1.65 min) than conventional method of laparoscopy (42.0 ± 2.69 min) for splenectomy in dog. Part of this difference might be due to the use of ultracision harmonic scalpel for sealing and cutting of the vessels and splenic attachments. Also, the experience of the surgeons has great impact on the outcome and operative time of this study. In one study on splenectomy using conventional laparoscopy in dog, the operative time was quite long in conventional three portals laparoscopy (115 ± 13.4 min) compared to open surgery (50.2 ± 6.6 min; 11). Apparently, experience of the surgeon could explain, in part, a long operative time. In other words, laparoscopic surgical times and complications tend to decrease with an increase in the level of the surgeon's experience, denominated learning curve [25-27].

The most time consuming part of the laparoscopic splenectomy is the time dedicated to remove spleen from the abdomen, which may be associated with the rupture of spleen. In the present study, 4 dogs (SILS: 3 dogs; Conventional: 1 dog) had slight and superficial rupture of spleen with negligible minor bleeding. This was managed successfully but elongated the surgical time.

## Conclusion

The single incision laparoscopic surgery (SILS) could be an available, feasible and safe alternative to multiport laparoscopy for dogs undergoing elective splenectomy. It presented advantages in relation to operative time and surgical scar, without any particular complication. The use of the appropriate vessel sealer to ensure hemostasis would facilitate the procedure and decreases the operative time.

## Methods

### Animals

This study was approved by the ethical committee of the Faculty of Veterinary Medicine, University of Tehran (BNS717/25.07.2009). Mongrel dogs (n = 18), weighting 15 ± 3 kg, were collected from Dog’s Shelter House, Animal Welfare Society. Experimental dogs were kept in individual pens and received standard balanced diet throughout experiment. Dogs were returned to the Shelter House after experiment.

### Anesthetic procedures

Following 8 hours food restriction, experimental dogs received acepromazine (0.1 mg/kg; IM) and buprenorphine (10 μg/kg; IV) for premedication and the combination of ketamine (5.5 mg/kg; IV) and diazepam (0.2 mg/kg; IV) for induction of anesthesia. The anesthesia was maintained by inhalation of isoflurane and oxygen through anesthetic machine. Cefazolin (22 mg/kg; IV) was administered as a preoperative prophylaxis at the time of inducing anesthesia.

### Surgical procedures

**Figure 1:**
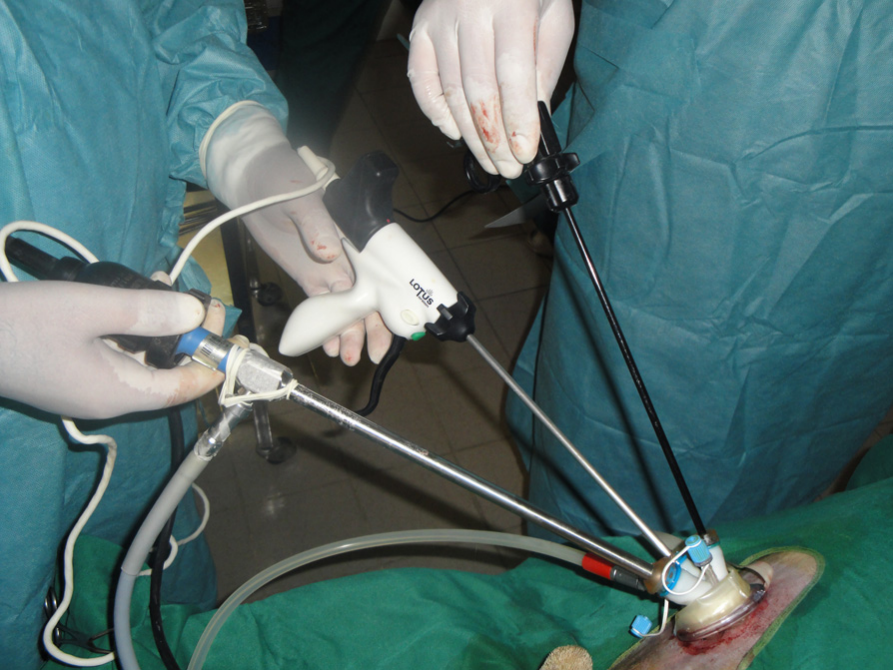
Single incision laparoscopic surgery (SILS).

**Figure 2:**
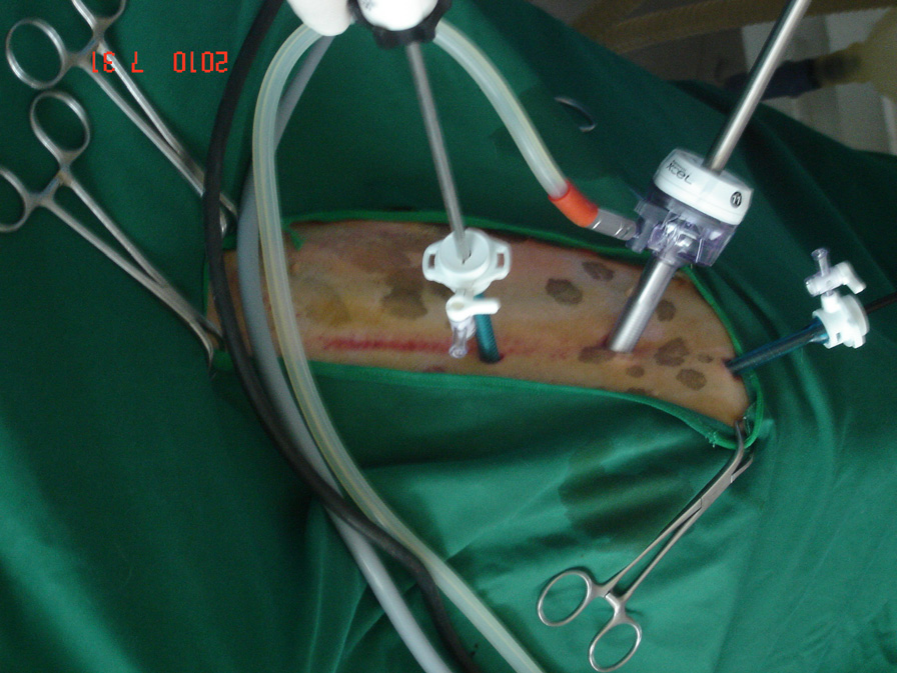
Conventional multiple portal laparoscopy.

**Figure 3:**
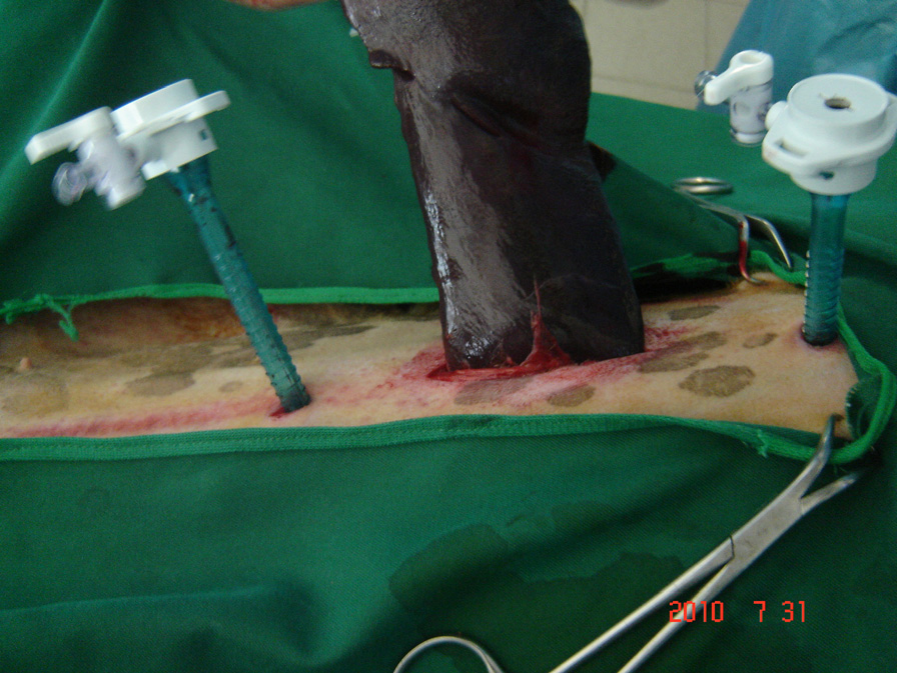
Removal of spleen from the abdomen.

The area from xiphoid to pubis was prepared under aseptic condition. Dog was placed in dorsal recumbency, head down and tilted 30 degree in the right lateral position. The surgeon and cameraman both stood on the right side of the dog and monitor was placed on the opposite side. For dogs operated by SILS method (n = 12), 3 cm midline skin incision starting from umbilicus to the caudal was made and the subcutis was reflected until revealing the linea alba. The linea alba was incised and the umbilical single portal was inserted using 5 mm TriPort trocar (Advanced Surgical Concepts, Wicklow, Ireland; Figure 1). For dogs operated by conventional method (n = 6), 3 portals including umbilical, cranial and caudal were inserted, 3 cm apart in a straight line, using 5 mm trocars. In both methods, the length of umbilical incision was enlarged, with routine surgical technique, according to the size of spleen (Figure 2). Pneumoperitoneum was established by CO_2_ using an automatic high flow pressure until the pressure of 12 mm Hg was achieved. A 5 mm in diameter 30 degree rigid telescope (29 cm length, Wolf, Germany) connected to a light source was inserted into the peritoneal cavity from the umbilical portal. The orientation of the spleen and the location of its proximal and distal poles were located initially. The 5 mm curved flexible-tip Maryland forceps (Carl Storz, Germany) was introduced from the cranial port and inserted through the splenic vessels at the hilum to lift the spleen up from its middle part or the more accessible portion. The ultracision harmonic scalpel (lotus hand piece, SRA Developments LTD, Devon, UK), was inserted from the caudal portal to seal and cut the splenic, left gasteroepiploic and short gastric arteries, veins and gastrosplenic ligament. The location of camera and forceps were changed during the operation to maintain ergonomy for better visualization and maneuver. The direction of sealing of the splenic vessels was highly depended on the size of spleen and its orientation. Thus sealing of vessels was started either from the center of spleen and continued cranially and caudally or from the distal pole and continued toward the splenic hilus to proximal pole. Following transection of the splenic attachments, the pedicles were checked to ensure hemostasis. With the help of camera from the enlarged umbilical portal, cranial pole of the spleen was oriented and removed. Then it was grasped by Doyen forceps and exposed through the incision. It was pulled out of the abdomen very firmly to avoid fracture of the splenic parenchyma (Figure 3). The abdominal incision was closed in a three layer routine manner. All surgeries were video recorded and the operative time, total length of the scar, weight of the spleen and its dimensions were estimated. Post operative complications were evaluated on Days 1, 3, 5, 7 and 30 after surgery.

### Statistical analysis

Data were analyzed using Student t-test after examining the assumptions of parametric tests using SAS/STAT [28]. Data were presented as Mean ± SE.

## Competing interest

Drs Alireza Khalaj , Jalal Bakhtiari , Amir Niasari-Naslaji as authors of this manuscript have no competing interests to disclose.

## Authors’ contributions

AK, JB designed the study and performed the study. JB drafted the manuscript. ANN carried out analysis and interpretation of the data and helped to draft the manuscript. All authors read and approved the final manuscript.
